# Factors affecting nursing students’ intention to use a 3D game to learn field triage skills: a structural equation modelling analysis

**DOI:** 10.1186/s12912-022-00826-0

**Published:** 2022-02-21

**Authors:** Meyrick C. M. Chow, Maria S. Y. Hung, JoJo W. K. Chu, Stanley K. K. Lam

**Affiliations:** 1 School of Nursing, Tung Wah College, 31 Wylie Road, Homantin Kowloon, Hong Kong; 2 Great Ormond Street Hospital for Children, Great Ormond Street, London, WC1N 3JH UK; 3 School of Nursing, LKS Faculty of Medicine, The University of Hong Kong, 4/F, William M.W. Mong Block, 21 Sassoon Road, Pokfulam, Hong Kong

**Keywords:** 3D games, Digital game-based learning, Technology acceptance model, Simulation, Healthcare education, Triage

## Abstract

**Background:**

As mass casualty incidents are low-probability events, students often do not have the chance to practise field triage skills during their clinical placement. This study used a 3D game to engage participants in experiential learning in a realistic virtual environment. The purpose of the study was to explore factors affecting nursing students’ intention to use a 3D game to learn field triage skills.

**Methods:**

This was a cross-sectional survey study. The technology acceptance model augmented by computer self-efficacy was used as a research model and a questionnaire was used to evaluate students’ intention to use the 3D game to learn field triage. Data was collected from nursing students of a degree-awarding higher education institution in Hong Kong.

**Results:**

A total of 177 valid questionnaires were returned, and structural equation modeling was used to test the research model and hypotheses. Consistent with the technology acceptance model, perceived usefulness (0.21, *p* < 0.05) and perceived ease of use (0.91, *p* < 0.001) had a positive effect on the behavioral intention to use the 3D game. Computer self-efficacy positively influenced both perceived usefulness (0.66, *p* < 0.001) and perceived ease of use (0.73, *p* < 0.001). The research model explained 42 percent of the variance in the behavioral intention to use the 3D game.

**Conclusion:**

Students believed that using the 3D game would enhance their field triage skills and found the game easy to use. Using 3D games to facilitate learning is a worthwhile educational approach for preparing healthcare professionals to handle low-probability clinical tasks, such as field triage in mass casualty incidents. Insights provided by findings of this study included the best way to design and promote interactive education programmes in a virtual environment.

**Supplementary Information:**

The online version contains supplementary material available at 10.1186/s12912-022-00826-0.

## Background

Accurate field triage is of great importance during disaster management. Foronda et al. [1] stated that nurses should understand the principles and be competent to perform triage, as it will affect them regardless of their role in disaster management. In the event of a disaster, emergency medicine (EM) personnel must be able to react accurately and swiftly. As such, Smith et al. [2] suggested that the main goal of nurse educators is to prepare nursing students to recognise problems of casualties at an early stage of triage, particularly those students who are in their final clinical experience. However, real clinical settings can be stressful and overwhelming, depriving students of learning opportunities as healthcare professionals strive to deliver vital care to casualties under time pressure, particularly in critical and emergency situations.

To enhance competency, a disaster-like simulation can be organised and planned. Disaster drills with standardised casualties are commonly used, but there are limitations due to various factors, such as expense and human resource management. As a result, these simulations are difficult, if not impossible, to repeat [1]. Virtual simulations can be an alternative. Healthcare professionals may use three-dimensional environments that simulate certain aspects of real-life scenarios, emphasising their dynamic and volatile qualities. Virtual reality (VR) has been adopted in clinical training across multiple contexts, such as disaster management, and is considered a viable cost-effective alternative solution for nurses to practise their skills [3].

VR is beneficial in training scenarios and that VR-based games allow learners to practice and assess their technical and conceptual skills as opposed to traditional methods [4, 5]. Evidence has shown that VR is a viable choice for training EM personnel in mass disaster triage, compared with standardised patient (SP) drills [6]. In addition, the greatest benefit of using VR is to provide trainees with a safe environment to practise their skills [7, 8]. Learners’ skills eventually improve and remain high through repeated use because they know what strategies to adopt [9]. VR can also improve users’ problem-solving skills and enhance their reflection on practice [10]. Indeed, VR users reported higher levels of confidence in triage and perceived themselves as effective responders [1].

VR has another implication in learning. Dickey [11] suggested that the pedagogy of virtual simulation is based on constructivism, which emphasises that knowledge is constructed by participants rather than transmitted by teachers. Therefore, learners play an active role in the learning process [12]. Indeed, studies have suggested that virtual simulation is an effective pedagogy in nursing education, and students generally have positive attitudes towards and enjoy learning through virtual simulation [1]. Regardless of the advantages of VR, there is no evidence to support literature claims of acceptance as such of a VR game in an undergraduate nursing programme to facilitate the acquiring of field triage skills. Therefore, the aim of this research is to explore factors affecting nursing students’ intention to use a three-dimensional (3D) game to learn field triage skills.

### The 3D field triage game

Serious games are games designed for a primary purpose other than pure entertainment. To date, all serious games in emergency medicine including field triage management are designed for use in overseas countries. Their scenarios are not designed in accordance with the Distinct Command and Control System in Hong Kong, nor the Hospital Authority Civil Disaster Contingency Plan. Most importantly, the field triage coding and scoring systems are different from those currently used by the Hospital Authority of Hong Kong. In this study, we designed and developed a 3D field triage game that simulated a real mass casualty incident in Hong Kong that happened on 1st May 2008 – the tragic Sai Kung bus crash. The driver of the bus carrying 61 passengers lost control of his vehicle at the roundabout near Ho Chung in Sai Kung, upturned on its side, and collided into a noise barrier. The roof of the bus was subsequently crushed and many passengers were pinned under the seats. With 19 people killed and 43 injured, it was one of the worst traffic accidents to occur in Hong Kong [13].

The 3D field triage game revolves around the training of nursing students as triage officers in the bus crash situation. Having arrived at the scene, the student will be advised that he/ she is the first-responder at the scene, and told that the scene is safe to enter. The student will be required to make a quick assessment of the situation, and then proceed with the triage task. The status of every casualty can be assessed by direct visualization and clicking on icons to perform the appropriate medical checks, e.g., the response check, breathing check, and capillary refill rate check. After completing the necessary checks and assessment, the student assigns the priority according to the field triage system currently used by the Hospital Authority: (1) red category – top priority; (2) yellow category – second priority; (3) green category – walking wounded; and (4) black category – dead. Once tagged, the student continues onto other casualties.

Each scenario comprises twenty casualties, which are randomly selected from a pool of 200 casualties with different types of injuries and degrees of severity. If the student fails to complete the field triage within 20 min, the simulation will pause and the student may choose to retry or exit the simulation. Training with imposed time limitations for completing tasks encourages and prepares students to perform effectively in a stressful environment. It is commonly called stress exposure training and has been demonstrated to enhance the development of quality decision-making skills [14]. During the triage, a score is kept and the student can receive in-game feedback after triaging each casualty. The score indicates how well the student is performing and this adds a competitive factor to the exercise. When students complete the entire procedure in the simulation, their performance will be saved in a log file, which can be reviewed by both the student and teaching staff.

## Methods

This was a questionnaire-based cross-sectional study. Using a 3D game-based virtual world to learn field triage skills is an innovative teaching strategy in nursing education, and whether students accept the technology is of particular interest to this study.

### Research model and hypotheses

In this study, the technology acceptance model (TAM) was adopted as a theoretical framework for analysing students’ perceptions of the 3D game-based virtual world. Conceptualised by Venkatesh and Davis [15], TAM is useful, effective and reliable in explaining and predicting an individual’s acceptance of a specific technology. The model is the most influential, most tested, and best-operationalized with good reliability and validity of its instrument [16, 17].

The model has two important constructs, perceived ease of use and perceived usefulness, influencing the behavioural intention to use a particular technology. Perceived ease of use indicates the extent to which an individual is free from physical and mental effort by using a particular system, while perceived usefulness indicates the extent to which an individual’s performance is enhanced by using a particular system [18]. Perceived usefulness is one of the essential components of the behavioural intention to use technology. Previous studies have found the positive association between perceived usefulness and behavioural intention [16, 17]. Evidence has also indicated that perceived ease of use is a determining factor in the use of technology [8]. The less effort the learners spend, the greater their intention to use the technology. Besides, it has been demonstrated that perceived ease of use affects perceived usefulness [15]. Perceived usefulness and perceived ease of use are also influenced by other external variables, such as computer self-efficacy explored in this study.

The construct of computer self-efficacy [19] was added as one of the variables in this research to account for new insights gained from the literature review and hopefully increase the total variance explained by the model. Self-efficacy is based on the social cognitive theory developed by Bandura [20] and refers individuals’ belief of their capacity to perform a specific task. In the context of computer use, computer self-efficacy refers to the user’s perceived capability to use computers to complete a task [19]. Studies have shown that as an external variable, computer self-efficacy positively influences the usefulness of e-learning [21]. Chow et al. [8] also found that computer self-efficacy had a positive effect on the usefulness and ease of use of learning in Second Life.

**Fig. 1:**
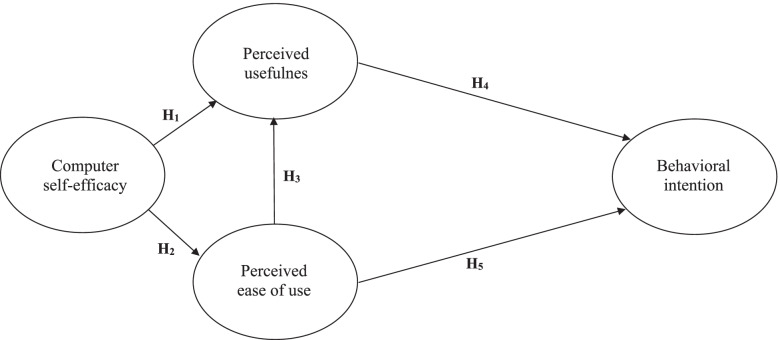
Research model and hypotheses. H_1_. Nursing students’ computer self-efficacy has a positive influence on the perceived usefulness of the 3D game-based virtual world to learn field triage skills. H_2_. Nursing students’ computer self-efficacy has a positive influence on the perceived ease of use of the 3D game-based virtual world to learn field triage skills. H_3_. Nursing students’ perceived ease of use has a positive influence on the perceived usefulness of the 3D game-based virtual world to learn field triage skills. H_4_. Perceived usefulness has a positive influence on the behavioural intention to use the 3D game-based virtual world to learn field triage skills. H_5_: Perceived ease of use has a positive influence on the behavioural intention to use the 3D game-based virtual world to learn field triage skills

There is a lack of quantitative evidence supporting the use of TAM in 3D game-based virtual world. Besides, it is meaningful to adopt this theory in the healthcare field for critical examination, as the results of testing the application of TAM in various healthcare professionals were not consistent [22, 23], and its application in nursing students was seldom explored. The aim of this study was to explore factors affecting nursing students’ intention to use the 3D game-based virtual world for learning field triage skills. The research model with five hypotheses is depicted in Fig. 1.

### Instrument

This study adapted items from validated instruments of published research studies (See the supplementary material). Computer self-efficacy and behavioural intention to use the 3D game-based virtual world to learn field triage skills were adapted from previous applications of TAM by Compeau and Higgins [19] and Chow et al. [8] respectively. Perceived ease of use and perceived usefulness were operationalized using an instrument developed by Venkatesh and Davis [15]. A seven-point Likert scale from (1) “strongly agree” to (7) “strongly disagree” was used to measure items of all the constructs.

### Sample and data collection

We commenced data collection after obtaining ethical approval. Undergraduate nursing students who were studying the course Trauma and Disaster Nursing at the School of Nursing of a tertiary institution were the survey sample. These students were chosen since they had the knowledge to triage casualties presented in the 3D game. As one of the learning activities of the course, students were arranged to participate in workshops on using the 3D game-based virtual world to learn field triage skills in computer laboratories. Such an arrangement would minimise self-selection bias, which might result if we only invited those who were interested to join the workshops. An introduction to manipulation and interaction in the virtual environment was arranged in the workshops to prepare the students who might be new to 3D game-based virtual world. Students were asked to play the 3D game after exploring the virtual world by themselves. Each workshop lasted for 2 h and upon completion of the workshops, we invited the students to fill in the questionnaire. All participants (*N* = 177) returned the questionnaire and they were between 20 and 28 years of age, and as expected most of them were female (72.9%). In terms of experience with 3D games, only a small percentage of participants had previous experience with 3D games (*N* = 55; 31.1%). In comparison, more than half of the participants had no experience with 3D games (*N* = 122; 68.9%).

### Statistical analysis

As recommended by Anderson and Gerbing [24], the measurement model was validated by conducting a confirmatory factor analysis (CFA), while the structural model was used for hypothesis testing. This study used the Amos 26.0 software for structural equation modelling (SEM) analysis.

## Results

Results of descriptive statistics of both the constructs and the individual item were summarized in Table 1. Students perceived that the 3D game was easy to use, useful and they were confident to use the 3D game proficiently and intended to use it whenever needed.

**Table 1:** Descriptive statistics, average variance extracted (AVE), composite reliability (CR) and factor loading of construct measurement

Variables	Mean^a^	Std Dev	Factor loading	t-value	AVE	CR
*Computer self-efficacy*	2.82	1.30			0.85	0.91
CSE1	2.84	1.25	0.87	17.83		
CSE2	2.79	1.34	0.96	17.83		
*Perceived usefulness*	2.24	0.95				
PU1	2.24	1.15	0.89	17.74	0.77	0.94
PU2	2.30	1.14	0.90	17.74		
PU3	2.20	1.07	0.90	17.41		
PU4	2.20	1.11	0.90	17.59		
*Perceived ease of use*	3.23	1.43				
PEOU1	3.24	1.57	0.88	7.86	0.57	0.85
PEOU2	3.40	1.69	0.88	7.88		
PEOU3	2.86	1.23	0.69	8.37		
PEOU4	3.41	1.24	0.56	8.37		
*Behavioral intention to use*	2.74	1.30				
BI1	2.67	1.27	0.94	18.37	0.83	0.92
BI2	2.81	1.32	0.90	18.37		

^a^1 – strongly agree and 7 – strongly disagree

### Measurement model

The fit indices in CFA showed that the measurement model was valid, since all of them were above their respective common acceptance levels (X^2^/df ratio = 1.75; Comparative Fit Index (CFI) = 0.98; Normed Fit Index (NFI) = 0.96; Non-Normed Fit Index (NNFI) = 0.97; and Root Mean Square Error of Approximation (RMSEA) = 0.07) [25]. Thus, the proposed model generally fits the sample data reasonably well.

We further assessed the psychometric properties of the measurement model. Regarding reliability, the composite reliabilities (CR) of the constructs are given in Table 1. All of the composite reliabilities exceeded the acceptable criterion of 0.6, which indicated a good reliability level [26]. Concerning convergent validity, we used the factor loadings of items and average variance extracted (AVE) of the constructs as indicators. Table 1 shows that both the factor loadings and AVEs of all items exceeded the benchmark of 0.50 as recommended by Hair et al. [27]. As for discriminant validity, Table 2 depicts that square root of the AVE of all constructs was larger than the correlation coefficient of each construct with other constructs, which was an indication of good discriminant validity [28].

**Table 2:** Square root of average variance extracted (AVE) and correlations of all constructs

		1	2	3	4
1	Computer self-efficacy	**0.92**			
2	Perceived usefulness	0.71	**0.88**		
3	Perceived ease of use	0.73	0.55	**0.76**	
4	Behavioral intention	0.82	0.69	0.67	**0.91**

Square roots of AVEs are shown as diagonal elements in bold type. The diagonal elements were greater than the corresponding off-diagonal elements in the same row and column, indicating the discriminant validity

### Structural model

**Fig.2:**
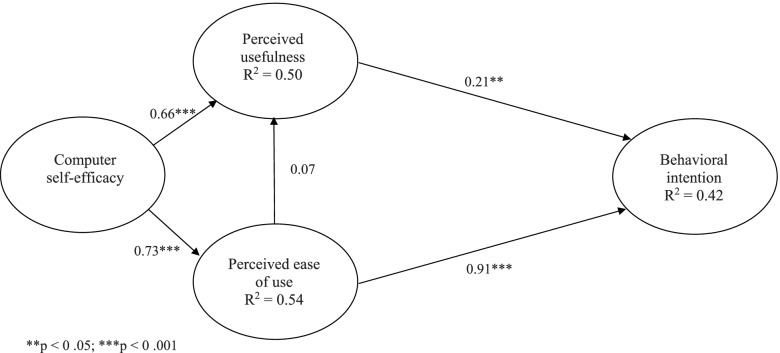
Results of structural equation model testing

Findings in Fig. 1 provided evidence of a good model fit of the structural model (*X*^*2*^/df ratio = 1.79; CFI = 0.98; NFI = 0.96; NNFI = 0.97; RMSEA = 0.07). Figure 2 shows the results of hypothesis testing, which was performed within the context of the structural model. Computer self-efficacy significantly influenced perceived ease of use (beta = 0.73, p < 0.001) and perceived usefulness (beta = 0.66, *p* < 0.001), hence hypotheses H_1_ and H_2_ were supported. Computer self-efficacy explained 54 and 50 percent of the variance in perceived ease of use and perceived usefulness respectively. However, perceived ease of use had no significant effect on perceived usefulness. Therefore, H_3_ was not supported. As expected, perceived ease of use (beta = 0.91, *p* < 0.001) and perceived usefulness (beta = 0.21, *p* < 0.05) had a significant positive effect on behavioral intention to use the 3D game, hence H_4_ and H_5_ were supported.

The research model explained 42 percent of the variance in the behavioural intention to use the 3D game in virtual world to learn field triage skills. Regarding the total effects, perceived ease of use was the strongest predictor for behavioral intention to use (0.94), followed by computer self-efficacy (0.82) and perceived usefulness (0.21). Additionally, computer self-efficacy had a strong indirect effect on behavioral intention to use (0.82). Table 3 summarized the direct, indirect, and total effects of computer self-efficacy, perceived usefulness, and perceived ease of use on behavioral intention to use.

**Table 3:** The direct, indirect, and total effects of variables on behavioral intention to use

	**Direct effect**			**Indirect effect**			**Total effect**		
	PU	PEOU	BI	PU	PEOU	BI	PU	PEOU	BI
CSE	0.66	0.73		***0.05***		0.82	0.71	0.73	0.82
PU			0.21						0.21
PEOU	***0.07***		0.92			***0.02***	***0.07***		0.94

*CSE* computer self-efficacy, *PU* perceived usefulness, *PEOU* perceived ease of use, *BI* behavioral intention to use

## Discussion

This study adopted well-validated scales to measure the constructs and used SEM analysis to test the model. Previous empirical studies of TAM have shown that perceived ease of use has a comparatively small effect on behavioural intention [15, 17]. In contrast, our study showed that perceived ease of use had a significant influence on the behavioural intention to use, as evidenced by the standardized path coefficient of 0.91 at the level of *p* < 0.001. This may indicate that being user-friendly is an important feature for students to use the 3D game. Since most of the participants had no experience with 3D games, if they found that the technology was complex and hard to use, it was very likely that they would reject the technology.

Consistent with previous results, computer self-efficacy was considered one of the significant variables determining perceived ease of use and perceived usefulness [29]. Our study found that computer self-efficacy explained 54% of the total variance in perceived ease of use and 50% of the total variance in perceived usefulness. People with high computer self-efficacy tend to have higher expectations of using a computer [30]. In short, students who had great confidence in using a computer found this system easy to use and useful. This can be explained by the motivation theory that considers computer self-efficacy as an intrinsic motivation factor and by Bandura’s social motivation theory [20], which suggests that high self-efficacy leads to an active learning process.

In our study, high self-efficacy had a greater influence on perceived ease of use, the regression weight being 0.73 at the level of *p* < 0.001, compared with the perceived usefulness of the 3D game. Therefore, increasing students’ confidence in computers will very likely positively affect their perceived ease of use of the 3D game. In addition, when promoting this e-learning method to students, one of the important foci should be on strategies that can enhance their computer self-efficacy, instead of solely concentrating on ease of use and usefulness. In a randomised trial, it was found that collaborative learning improved the students’ learning achievements and awareness of problem-solving [31]. In collaborative learning environments, participants work in small groups and the learning process is enhanced through interacting and discussing the knowledge resources and information that they are all aware of. The explicit collaboration of the group members on activities such as how to handle the task and when to include specific resources can effectively enhance their computer self-efficacy and persistence in using educational 3D computer games. Moreover, positive persuasion, such as giving constructive and positive feedback, can increase students’ confidence, which is one of the essential components of computer self-efficacy [31].

In contrast to previous empirical studies of TAM [6, 8], our study revealed that perceived ease of use had no effect on perceived usefulness. According to Johnson and Payne [32], usefulness can be defined by ‘benefits’ and ‘cost’, while ease of use can be viewed as the cost of using the system from the user’s perspective. Therefore, if a person finds the system easy to use, it may have a positive effect on its perceived usefulness. Yet, many of our nursing students were quite neutral on ease of use of the 3D game, as revealed by an indifferent mean score of 3.23 on the construct. This may be explained by the difficulty of using the keyboard and the mouse in playing the 3D game. Another possible explanation may be the difficulty of using the software due to a lack of experience. The descriptive statistics indicated that nearly 70% of participants had no experience with 3D games. Although students participated in a short workshop before starting the game, it was still a relatively new experience for them as learning to use a new tool takes time. It is expected that extending the learning time with the new tool will have an effect on ease of use. Gender can also be one of the determinants of perceived ease of use. Ong and Lai [21] found that men tended to score higher in perceived ease of use compared with women. Similarly, women placed more importance on ease of use than men. Although gender was not adopted as one of the exogenous variables in this study, women were the dominant group, accounting for three quarters of the participants. Overall, the insignificant relationship between perceived usefulness and perceived ease of use may be due to insufficient experience, difficulty in using the system and gender differences. Consequently, it is recommended to organise a long introductory session and provide adequate technical support in case of difficulty. In addition, future studies should consider the use of touch screens or increase the speed of display of graphics, which may contribute to ease of use.

Compared with perceived ease of use, perceived usefulness had relatively less effect, but was statistically significant, on the behavioural intention to use the 3D game, as shown by the standardised path coefficient of 0.21 (*p* < 0.05). Despite the difficulty of using this system and the voluntary nature of participation in this study, our study found that usefulness was an influential construct. Many nursing students may not be involved in field triage during their clinical placement. Findings of previous studies have shown that many nurses are not well prepared to participate in disaster management and tend to have higher levels of anxiety in field triage [6]. Conversely, this 3D field triage game can provide a safe environment in which students are fully immersed to practice their skills and enhance their knowledge without endangering patients’ lives. In addition, one of the distinct features of this 3D game is that it provides feedback immediately during and at the end of a session, without the presence of a teacher. Hence, students may find it interactive and constructive. Furthermore, students can freely access this game and repeat it unconditionally to meet their learning needs.

Our extended TAM model, together with computer self-efficacy, accounted for 42% of the total variance in behavioural intention to use. Although this value is relatively high, there are nearly 60% of the total variance caused by other unknown factors. Therefore, future studies may explore other constructs to strengthen the research model, and use qualitative research to provide an in-depth exploration of students’ perception on using the 3D game to learn field triage skills.

Arranging workshops on using the 3D game-based virtual world to learn field triage skills as one of the learning activities of the course Trauma and Disaster Nursing and with all students voluntarily completed the questionnaire at the end of workshops helped minimise self-selection bias. Limitations of the study included data collection in a single tertiary education institution, and focused only on nursing students’ perceptions. Other institutions and healthcare disciplines, such as physicians and paramedics, could also be studied to increase the generalisation of the results. Besides, we added one exogenous variable to TAM in this study. More factors might be included in future research to increase the predictive and explanatory power of TAM.

## Conclusion

This study used SEM to analyse the data and ensure methodological rigor by testing the measurement and structural models. TAM can be an effective model for predicting students’ intention to use the 3D field triage game. Overall, students’ perceptions of the 3D game were influenced by computer self-efficacy, perceived ease of use and perceived usefulness. In the future, studies employing different research methodologies are encouraged that may generate relevant and interesting findings in using 3D games to enhance healthcare education.

Accurate field triage is critical to recognise problems of casualties at an early stage of disaster management. In critical and emergency situations, healthcare professionals strive to deliver vital care to casualties under time pressure. Such stressful and overwhelming situations often deprive them of learning opportunities in real clinical settings. This study took a proactive and creative educational approach using a 3D game to engage participants in experiential learning in a realistic context and authentic setting. Promising findings of this study have indicated that using 3D game-based virtual environments is a viable option to enhance learning and increase learning motivation. This educational approach is particularly useful for preparing healthcare professionals to handle low-probability clinical tasks such as field triage in mass casualty incidents in this study.

## Supplementary Information

**Additional file1.**

## Data Availability

The datasets used and/or analysed during the current study are available from the corresponding author on reasonable request.

## Abbreviations

3D: Three-dimensional
TAM: Technology acceptance model
SEM: Structural equation modelling
CFA: Confirmatory factor analysis
CRI: Comparative Fit Index
NFI: Normed Fit Index
NNFI: Non-Normed Fit Index
RMSEA: Root Mean Square Error of Approximation
CR: Composite reliabilities
AVE: Average variance extracted
