# Supplementary material for: Factors affecting nursing students’ intention to use a 3D game to learn field triage skills: a structural equation modelling analysis
Source: BMC Nurs. 2022 Feb 21;21:46. doi: 10.1186/s12912-022-00826-0 (PMC8862333; doi:10.1186/s12912-022-00826-0)
Supplement: Supplementary file 1 — Additional file1. [file 12912_2022_826_MOESM1_ESM.doc]

**Supplementary material**

Survey items

| *Computer self-efficacy*  CSE1 I expect to become proficient in using *3D Field Triage Game*.  CSE2 I would feel confident that I can use *3D Field Triage Game.*  *Perceived usefulness*  PU1 *3D Field Triage Game* can improve my learning efficiency.  PU2 *3D Field Triage Game* can enhance my learning performance.  PU3 *3D Field Triage Game* increases my learning output.  PU4 I find *3D Field Triage Game* useful for my learning.  *Perceived ease of use*  PEOU1 It is easy to operate *3D Field Triage Game* and get it to do what I want it to do.  PEOU2 I find that *3D Field Triage Game* is very easy to use.  PEOU3 I find that the human interface of *3D Field Triage Game* is clear and easy to understand.  PEOU4 I find that interacting with *3D Field Triage Game* doesn’t demand much care or attention.  *Behavioral intention to use*  BI1 Given that I had access to *3D Field Triage Game*, I predict that I would use it.  BI2 I intend to use *3D Field Triage Game* as often as needed. |
| --- |
